# The effect of a commercial probiotic drink on oral microbiota in healthy complete denture wearers

**DOI:** 10.3402/mehd.v23i0.18404

**Published:** 2012-10-03

**Authors:** Justyna Sutula, Lisa Coulthwaite, Linda Thomas, Joanna Verran

**Affiliations:** 1 Microbiology Research Group, School of Health Care Science, Manchester Metropolitan University, Manchester, UK; 2 Yakult UK Ltd, Odyssey Business Park, Middlesex, UK

**Keywords:** Lactobacillus casei Shirota, Candida, denture wearers, denture biofilm, saliva, colonisation, OHIP, well-being

## Abstract

**Background:**

It is acknowledged that oral and general health status declines with age. The global population of denture wearers is increasing, so is the incidence of denture biofilm-related problems, such as denture-associated stomatitis, aspiration pneumonia and malodour. It has been suggested that consumption of probiotic bacteria may improve oral health. However, the effects of probiotics on the oral microbiota of denture wearers have received little attention.

**Methods:**

The aim of this study was to investigate the effect of consumption of a commercial probiotic product (Yakult) on microbiota of saliva, tongue and denture biofilm in healthy complete denture wearers. Eight healthy complete denture-wearing National Health Service (NHS) patients undertook a 7-week trial comprising three phases: baseline; 4-week consumption of one bottle of Yakult per day, each containing a minimum 6.5×10^9^ viable cells of *Lactobacillus casei* strain Shirota (LcS); 4-week washout period. The microbial viability and composition of saliva, tongue dorsum coating and denture biofilm were assessed using a range of solid selective and indicator media. Questionnaires were used to explore participants’ denture cleaning habits and impact of wearing dentures on their life quality and well-being [modified oral health impact profile (OHIP-14)] prior to and after the study.

**Results:**

Seven volunteers (1 male, 6 females) completed the trial. LcS temporarily colonised oral cavity and denture surface. There was no significant change in the viability of *Streptococcus mutans*, acidogenic microorganisms, total anaerobic species and Gram-negative obligate anaerobes between study phases. There was no obvious effect of LcS on occurrence and viability of *Candida*. Participants presented a good general knowledge of denture hygiene and their responses to OHIP-14 questionnaires improved after completing the study (*p*=0.16).

**Conclusion:**

It appeared that 4-week consumption of probiotic drink had no overall effect on selected oral parameters in healthy denture wearers despite temporary presence of LcS.

## Introduction

In edentulous individuals, acrylic prostheses (dentures) provide a hard non-shedding surface that facilitates bacterial adhesion and subsequent colonisation. The maxillary fitting plane tends to retain nutrients, which favours accumulation of acidogenic bacteria, particularly streptococci and lactobacilli. The likelihood of the presence of yeast is also increased (1–3). Colonisation of denture materials with *Candida albicans* is a known aetiological cause of denture-associated stomatitis (sore mouth), which affects 24–75% of denture wearers (4, 5). It has been recognised that the oral status of older people can impact on their general health, quality of life and well-being (6). It has been suggested that probiotic microorganisms, so far extensively studied for their beneficial effect on the intestinal flora (7, 8), could be used to maintain oral health (9). Probiotics are defined as ‘live microorganisms which when administered in adequate amounts confer a health benefit on the host’ (10). Adhesion and colonisation of denture materials by probiotic bacteria are possible mechanisms to interfere with binding of pathogens through competition for adhesion sites, nutrients and alteration of the local environment. However, there is a limited understanding of the relationship between dental materials and probiotic consumption (11). Furthermore, to the authors’ best knowledge, this is the first study investigating simultaneously the effect of probiotic consumption on oral health and oral health-related quality of life of complete denture wearers.

The aims of this study were to investigate the effect of 4-week consumption of a commercially available probiotic fermented milk drink (Yakult) on the microbiota of saliva, tongue, denture biofilm and the ratings of modified oral health impact profile (OHIP-14) in healthy full-denture-wearing individuals.

## Materials and methods

### Study design

NHS patients attending their scheduled appointments at a local dental hospital were approached by a clinician to participate in the oral health study. A member of the research team assessed suitability for the study criteria. Those who fitted the criteria and initially agreed to participate were handed over an information pack containing the participant information sheet, consent form, leaflet with guidelines on denture cleaning and probiotic usage and a questionnaire. Participation was voluntary and informed; signed and witnessed consent was obtained on the second visit prior to sampling. Ethical approval for the study was obtained from NHS Research Ethical Committee (LREC reference number: 08/H1011/72).

The intervention study comprised a maximum of five visits with samples of saliva, tongue and denture biofilm being obtained every 2 weeks during normal dental appointments at the clinic. Baseline levels of microorganisms were obtained during the first sampling visit (participants acted as their own controls), which was followed by 4 weeks of consumption of a probiotic fermented milk drink (28 day supply, one bottle per day, Yakult) and 4-weeks of washout, during which participants stopped taking the product but continued the microbiological sampling.

### Participation criteria

Participants were self-proclaimed healthy men and women aged 55 years and above, edentate individuals wearing both dentures (upper and lower), without pre-existing oral irritations. Smokers (our previous attempted recruitment of NHS denture-wearing patients revealed that around 50% of the patient population were smokers; unpublished results) and individuals with mild and controlled diabetes were included. History of intra-oral surgery within the last 6 months was noted. The exclusion criteria were pregnancy, oral antibiotics within the last month, use of a chlorhexidine mouthwash within the last month, lactose intolerance, vegan diet, systemic diseases and poorly controlled diabetes.

Throughout the trial, participants were asked to refrain from the consumption of medicated sweets and other probiotic-containing products, and to limit yoghurt intake to a maximum of four standard 120 g pots per week. Subjects were asked to refrain from oral and denture hygiene in the morning prior to sampling. Mechanical tongue scraping practices were also prohibited. A leaflet containing normal denture hygiene procedures recommended by the British Health Foundation (2008) was developed and distributed for participants to adhere to throughout the study. The denture cleaning steps included brushing both dentures (upper and lower) with a soft toothbrush to remove any food debris and loose plaque, followed by soaking in a denture cleaning product. The next step included further brushing and rinsing under running water.

### Probiotic product

Participants were provided with 28 days’ supply of the commercial probiotic fermented milk drink, Yakult (supplied by Yakult UK Ltd). According to the product label, each 65 ml bottle of the fermented milk drink contained a minimum of 6.5×10^9^ viable cells of *Lactobacillus casei* Shirota (LcS). The viability tests performed in our laboratory on three randomly selected bottles from separate batches confirmed a high viable count of 1.1×10^10^ ± 1.2×10^3^ cells per 65 ml bottle. Participants were advised to consume one drink per day, preferably at the same time point, for example, after a meal, with complete denture inserted in their mouth. Participants were also advised to reduce the pace of consumption of the drink, to take small sips to increase contact time between oral surfaces and the probiotic bacteria. A leaflet containing information on handling and storage of the product was also provided to the participants.

### Sampling

Sampling of oral health parameters was performed every 2 weeks during scheduled appointments at the clinic. Samples of saliva, tongue and denture biofilm were obtained from participants prior to any procedures performed by clinical staff, such as oral and denture examinations. All sampling techniques were demonstrated to participants and step-by-step guidelines with illustrations were presented.

Unstimulated whole saliva (approximately 2 ml) was collected by participants themselves by expectoration into a sterile 25 ml plastic bottle that was kept on ice until processing (within 2 hours of collection). One ml of the stock was serially diluted in sterile phosphate buffered saline (PBS) 10-fold to 10^−5^ (CFU ml^−1^) and the remainder was stored neat at −20°C. Tongue biofilm of a known sampling area was collected by participants themselves using a commercially available soft bristle toothbrush, previously sterilised by autoclaving at 121°C for 15 min. Participants obtained samples by rocking the brush gently against the dorsal region of the tongue for 5 seconds, avoiding triggering the gagging reflex. The biofilm was then aseptically suspended in reduced transport fluid (RTF). In the laboratory, the biofilm suspension was serially diluted in PBS to 10^−3^. Contaminated toothbrushes were placed in sodium hypochlorite solution (1:40) for 10 min, then washed in distilled water twice and autoclaved on the waste cycle. This was followed with a second soak in distilled water. After drying, toothbrushes were prepared for sterilisation and reuse as already described. Each participant had his/her own toothbrush throughout the study.

Complete dental prostheses (maxillary and mandibular arch) were collected from participants into plastic containers and taken away to be imaged by digital white light photography (Sony Cyber-shot DSC-T9 digital camera, 6MP, 3×optical zoom) and sampled prior to any manipulation by clinical staff. The fitting surface of both upper and lower denture was sampled by rubbing a sterile alginate swab previously moistened in sterile pre-reduced RTF, back and forth ten times over the entire denture fitting area. The swab was placed in 5 ml of sterile pre-reduced RTF and stored under anaerobic conditions (anaerobic jars with AnaeroGen sachet) at 4°C until processing. Sampled dentures were disinfected in 0.1% sodium hypochlorite solution for 15 min, brushed with a commercially available soft bristle toothbrush previously sterilised by autoclaving at 121°C for 15 min and rinsed under running tap water before returning to the patient. In the laboratory, the swabs were vortex mixed for 2 min (4×30 seconds) and the resultant suspension was serially diluted in PBS to 10^−3^.

### Microbiological analysis

Serial dilutions of saliva, tongue and denture biofilm were plated out onto selective and indicator media for estimation of viable count (colony forming units per ml of saliva; per cm^2^ of sampled tongue and denture fitting area per ml) of specific microorganisms and indicator microbial groups. Microbial numbers were obtained and selected colonies were subcultured for preliminary identification tests. Solid media used were ‘LcS select’ agar, which was developed in our laboratory for selective isolation of the probiotic strain *L. casei* Shirota (LcS) from mixed culture (12). The formulation was based on de Man Rogosa Sharpe medium (Oxoid) additionally supplemented with vancomycin and bromophenol blue pH indicator to facilitate selective isolation and differential colony morphology development, characteristic of LcS. Plates were incubated in CO_2_ enriched atmosphere at 35°C for 72 hours. Total facultative and obligate anaerobes were isolated on fastidious anaerobic agar (FAA; LabM, Bury, UK) supplemented with 5% defibrinated horse blood. Gram-negative anaerobic species, including black pigmented anaerobes were isolated on FAA supplemented with G-N Anaerobe Supplement (GNA; Oxoid). Both FAA and GNA media were pre-reduced before inoculation and incubated at 37°C anaerobically (80% N_2_, 10% H_2_, 10% CO_2_) for 5 days. Acid-producing organisms were explored using bromocresol purple indicator agar (BCP) incubated at 35°C in 5% CO_2_ for 24 hours (13). BCP indicates acid production, with pH range of 5.2 (yellow) to 6.8 (purple). A yellow halo around a colony on a purple agar indicated an acidogenic colony. The acidogenic ratio was calculated as the total number of acid-producing colonies/total number of colonies and expressed as a percentage. Sabouraud dextrose agar (SABC; Oxoid) supplemented with chloramphenicol (LabM) was used for isolation of yeast, incubated aerobically at 37°C for 3 days. Mitis Salivarius agar (Difco™; Becton, Dickinson and Company, France) with 1% potassium tellurite solution (BBL) and bacitracin (0.2 units ml^−1^; Sigma) was used for isolation of *Streptococcus mutans* species, which produce characteristic blue colonies with granular ‘frosted glass’ appearance (14, 15). Plates were incubated anaerobically at 37°C for 3 days.

### Questionnaires

#### General questionnaire

Individuals who agreed to participate in the study received a set of 16 multiple choice ‘tick box’ questions, previously prepared by the research team. Patients were required to select answers most appropriate to duration of denture experience; denture cleaning and storing habits; knowledge of products used for denture hygiene and source of information on denture hygiene. Patients were allowed to select more than one answer to multiple choice questions. A number of questions were open-ended, giving the patients opportunity to enter individual responses. In addition, the questionnaire explored patients’ demographic characteristics, namely, gender and age range.

#### Modified OHIP-14

OHIP-14 is a 14-item questionnaire designed to measure self-reported functional limitation, discomfort and disability attributed to oral conditions (16). It is a shortened version of original set of 49 questions (17). The original questionnaire was based on theoretical model of oral health developed by Locker (18) and a framework formulated by the World Health Organization (19) to classify general health-related impairments (biological loss of structure or function), disabilities (functional limitation, discomfort on behavioural level) and handicaps (social constraints). In the context of oral health, tooth loss resulting from oral disease (impairment) can lead to difficulties in chewing (functional limitation) or can cause pain (discomfort). This in turn may lead to a restricted ability to eat and the need to alter diet (disability). In severe cases, social isolation (handicap) brought on by all the above factors may result.

A modified version of the OHIP-14 questionnaire was distributed to explore the frequency of problems experienced by patients with their mouth or dentures in the past 12 months (16, 20). The modified version used in the study contained two additional questions (to give a total of 16 questions) regarding perception of malodour and denture odour. These criteria are not included in any other oral-health-related quality of life (OHR-QoL) questionnaire, although they have been proposed to be ill-defined problems (21, 22), which ultimately may impact QoL. Patients were asked to rate their experience of dental problems using a 5-point Likert-type scale coded 1 (never), 2 (hardly ever), 3 (occasionally), 4 (fairly often) and 5 (very often). OHIP-14 impairment was characterised by the summary score, that is, the sum of the 16 scores, where high scores indicated poor OHR-QoL and low scores indicated satisfactory OHR-QoL. To investigate any differences in perceived oral-health-related problems, participants were asked to complete the questionnaire before and after study.

Both questionnaires were completed by all participants in their leisure time. The questionnaires were anonymised and identified using a reference number allowing linkage between them and microbiological data from each participant. All the answers to questions were coded and entered into a spreadsheet for analysis.

### Analysis

The numbers of yeast and bacteria in saliva, tongue and denture biofilm were compared to indicate any possible correlations. The statistical significance of the relationships was determined using Pearson's population correlation coefficient in Minitab (version 15). The changes in microbial numbers over the study periods were analysed to investigate the effects (if any) of the probiotic on the oral microbiota. General questionnaires were analysed for frequency of responses.

Sixteen responses to modified OHIP-14 questionnaire (pre- and post-study) were converted into numerical scores, that is, never = 1, hardly ever = 2, occasionally = 3, fairly often = 4 and very often = 5. For each completed questionnaire, a summary score was calculated using the additive count method (23) by summing the item codes for all 16 items, irrespective of frequency. The summary score range was 16–80.To identify the most frequently reported oral-health-related problems, a number of negative impacts reported as experienced occasionally, fairly often or very often was counted across all 16 items and compared before and after study (16).

The significance of changes in responses given prior and post probiotic study was tested with paired sample *t*-test, comparing mean scores with the level of significance set to 5%.

## Results

### Response

A total of 63 patients (33 males, 30 females) were approached during their scheduled appointments at the dental hospital over a period of 2 weeks. On assessing patient suitability for the purpose of the study, 26 individuals fitted the inclusion criteria. Of those, only eight agreed to participate (one male, seven females). The main reasons for failed recruitment were: presence of partial denture or remaining teeth (25%), missing denture (maxillary/ mandibular or both), unworn, ill-fitted set (16%), poor general health, such as systemic conditions and severe diabetes (8%), and age below 55 years (10%). Four of the patients approached reported diagnosed lactose intolerance or proclaimed allergy to dairy products, and seven were not willing to participate. In total, seven participants completed the trial (one male, seven females; age 59 and 76 ± 11 years, respectively). One participant from the recruited group was prescribed an antibiotic treatment during the first phase of the study and therefore was excluded from further investigation. All remaining participants were sampled on at least four occasions, every 2 weeks during their appointments.

### Denture analysis

**Fig. 1:**
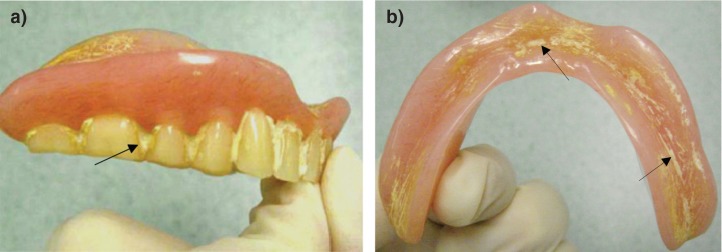
Heavy encrustation of calculus and plaque (arrowed) on maxillary (a) and mandibular dentures (b). The maxillary and mandibular dentures present a heavily abraded fitting area with deposited calculus and stains.

In general, the majority of inspected dentures (seven sets) were clean and in a good condition. Three out of seven sets had visible plaque between teeth, with accompanied yellow staining. One set of denture prostheses showed heavy deposits of calculus between teeth (Fig. 1a). The maxillary denture was covered with white calcified deposits. The mandibular denture showed signs of scratches covered with stains and calculus (Fig. 1b). None of the sampled dentures produced any noticeable unpleasant odour. Some dentures had retained food residue.

### Microbiology

Overall, 4-week consumption of one bottle of probiotic fermented milk drink (Yakult) containing a minimum of 6.5×10^9^ viable cells of LcS per day did not significantly affect the microbiota of saliva, tongue or denture biofilm in investigated individuals. Similar findings were reported by Sutula et al., (12), describing no significant effect of Yakult consumption on the oral flora of healthy dentate individuals.

Presumptive LcS was present in saliva of six out of seven participants during the intervention phase. Out of them, four participants also carried the probiotic strain on the dorsal region of the tongue. The bacterium was found on maxillary denture in three participants and on the mandibular denture in one during the probiotic consumption phase. The average concentration of LcS during the intervention phase was 8.1×10^3^ (± 8.5×10^3^) CFU ml^−1^ of saliva; 1.1×10^3^ (± 9.5×10^3^) CFU cm^−2^ of tongue biofilm; maxillary denture biofilm 1.2×10^3^ (± 1.0×10^3^) CFU ml^−1^; mandibular denture biofilm 2.5×10^2^ (± 2.1×10^2^) CFU ml^−1^. The isolation of LcS decreased or reduced to zero in saliva and tongue biofilm after discontinuation of the probiotic consumption. The probiotic strain was recovered from the maxillary denture fitting surfaces of two individuals up to 7 weeks after washout at concentration of 1.5×10^2^ (± 7.1×10^1^) CFU ml^−1^ and from the mandibular denture in one of the two participants (1.5×10^2^ ± 7.1×10^1^ CFU ml^−1^).

There was no significant change in the average number of facultative and obligate anaerobic species (FAA counts) and obligate anaerobic bacteria (GNA counts) isolated from saliva, tongue and denture biofilm throughout the study. The counts fluctuated throughout the trial but no trend was found. No black pigmented anaerobic species were recovered from any sample during the study.

Presumptive *S. mutans* species were found sporadically in saliva of five participants at a low concentration up to 1.0×10^2^ CFU ml^−1^ in 1: 10 dilution (levels obtained below the 25–250 colonies countable range). None were isolated from tongue or denture plaque. The percentage of acid-producing microorganisms isolated from saliva of all participants remained relatively stable during the trial. Fluctuations in the acidogenic ratio of tongue plaque bacteria did not always reflect the changes in saliva. There was no clear relationship between the acidogenicity of saliva and tongue plaque and denture plaque or between acidogenicity and probiotic consumption.

At baseline, no participants carried yeast in the oral cavity or on sampled denture surface. After 2 and 4 weeks of the probiotic consumption, the yeast genus, *Candida*, was isolated from saliva of three participants at low levels 1.0×10^2^ CFU ml^−1^ in 1: 10 dilution and remained at this level up to 6 weeks of washout. *C. albicans* was isolated from saliva from two individuals. Individuals tended to harbour more than one species of yeast but always at very low numbers. Other identified *Candida* species included *Candida tropicalis*, *Candida guilliermondii* and *Candida parapsilosis*. Single colonies of *Rhodotorula* species were isolated from saliva of two participants.

### Questionnaires

#### Responses to general questionnaire

Recruited patients (one male and six females) completed the general questionnaire prior to taking part in the study. Participants reported wearing denture prostheses on average for 28 ± 22 years, due to tooth loss caused mainly by periodontal disease, and had been wearing their current denture set for 7 ± 7 years. When asked about expected denture lifespan, the majority of patients (six out of seven) responded that it depends on an individual, but three patients also indicated time-scales: up to 5 years, 5–10 years and over 10 years, respectively.

All participants, except for one, removed dentures to sleep. The most often used method for storing dentures while out of the mouth was a glass of water (*n*=6). All questioned participants reported that they removed their dentures for cleaning, at least once or twice per day (*n*=4 and 3, respectively); always the first thing in the morning (*n*=7), the last thing at night (*n*=2) and after meals (*n*=1). From the choice of denture cleaning methods provided in the questionnaire, those most often selected in descending order were: denture brush, water rinse/soak (*n*=4); denture paste, denture tablet (*n*=3); toothpaste, denture cleansers containing 1.5–2% hypochlorite (*n*=2). The most frequently noted denture hygiene methods/products were toothpaste and denture tablet (*n*=5) and the main source of information about denture hygiene maintenance was a dentist (*n*=6).

#### Responses to modified OHIP-14

**Fig. 2:**
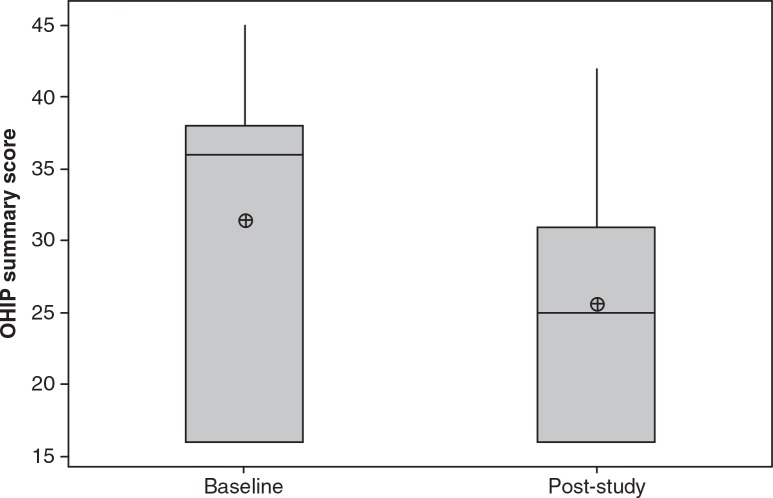
Boxplot of the modified OHIP-14 summary scores for complete denture wearers (*n*=7) assessed before (Baseline) and after completion of probiotic study (post-study). Reduced post-study scores indicate improvement in responses (*p*=0.16). The median is represented by the horizontal line. The mean value is represented by a crossed dot.

The OHIP-14 summary scores ranged between 16–45 at baseline and 16–42 after completion of the study (Fig. 2). The obtainable summary score range for the questionnaire was 16–80, where low scores indicated satisfactory oral-health-related quality of life and high scores were associated with poor life quality.

There was a decrease in the average summary scores obtained from the modified OHIP-14 questionnaires after study completion (25.6±9.2) in comparison to the baseline scores (31.4±11.2); however, no significant difference was found (*p*=0.16).

At baseline, the most frequently reported problems affecting patients occasionally, fairly often or very often were: finding it uncomfortable to eat (*n*=5), experiencing painful aching in the mouth (*n*=5), being a bit embarrassed (*n*=3) and feeling self-conscious about malodour (*n*=3). Following study completion, the most frequently indicated problem was finding it uncomfortable to eat, which was reported by three patients. Other highly scored items were reported at reduced severity levels after the study had finished but no significant difference was found.

## Discussion

It has been estimated that by the year 2020, almost half (48%) of the world population will be over 50 years old as a result of improved sanitation, hygiene, and healthcare in developed countries (6). This population is likely to wear dentures, since aging is associated with eventual edentulism and the need for dental prostheses (24). With the increase in the estimated lifespan of the human population, it is essential to direct more attention to the care of geriatric patients, especially debilitated ones, with regard to oral hygiene, due to increasing infections associated with yeast, and inhalation pneumonia associated with a range of opportunistic pathogens in denture plaque (25). There is increasing evidence to suggest that the elderly could particularly benefit from probiotic consumption (26, 27). Age-related changes of the gut microbiota make this group vulnerable to growth of pathogens and increased disease risk (28). It has been recognised that poor oral health and tooth loss in the elderly can impact on the level of nutrition intake, which in turn can affect general health and management of existing medical conditions (29). Edentulism has also been associated with a significant psychosocial impact, including psychological discomfort (feeling self-conscious, embarrassed, tense or anxious about the mouth or denture) and social handicap (irritability with others, inability to perform usual activities), which all can reduce the quality of life of an individual and ultimately their perception of well-being (20).

Few effects of various probiotic formulations containing lactobacilli, streptococci and bifidobacteria have been studied in dentate population in terms of potential reduction of cariogenic bacteria, improvement of gingival health and antimalodour effect (30–32, for the review see 33). LcS is one of the most studied probiotic strains with scientifically proven gastrointestinal system health benefits (34). Despite that, its effect on oral health parameters has received very little attention. To the best of authors’ knowledge, there are two studies describing use of the LcS-containing product, Yakult, on gingival inflammation in dentate individuals (35, 36) but there has been no intervention study investigating the effect of this product on oral microbiota in healthy edentulous complete denture wearers.

In this study, the transient presence of LcS in saliva and tongue plaque during the probiotic intervention phase was demonstrated (*n*=6) using the newly developed selective medium ‘LcS Select’. The probiotic strain was also recovered from the fitting surfaces of dental prostheses in three individuals.

After discontinuation of the probiotic consumption, numbers of the probiotic strain decreased and eventually reduced to zero in all sampled sites, suggesting intake-dependent temporary colonisation. This would support reports of the transient nature of this strain in the intestinal microbiota indicated by intervention studies (7). LcS can be detected in faecal samples only for a limited period after the consumption phase has finished (37).

In the present study, the probiotic strain was present on acrylic surfaces for up to 7-weeks of washout but not in tongue plaque or saliva in two individuals. It is likely that the LcS adhered and colonised the occluded fitting area of the denture during the consumption period. Perhaps the varied surface topography of the prostheses prevented the probiotic strain from being cleared and allowed viable occurrence after post-consumption period, albeit temporarily and in low numbers. Other authors have described transient manifestation of probiotics in dentate individuals (30, 38). A group in Finland not only reported up to 12 days retention of *Lactobacillus rhamnosus* GG (LGG) in the mouth after a 14-day consumption of 200 ml of juice containing 5×10^6^ CFU ml^−1^ of LGG but also identified an individual case of long-term colonisation in a female who was given LGG-containing milk for 1 year in her childhood (39). In a recent study by Caglar et al. (40) investigating oral colonisation of probiotic *Lactobacillus reuteri* 55730 following a daily exposure to 10^8^ CFU per tablet over a fortnight, a temporary and decreasing with time salivary occurrence was reported (up to 5 weeks in two individuals after discontinuation). Thus, despite differences in dose, frequency of consumption, duration of consumption or the particular probiotic strain used in *in vivo* trials, no permanent establishment on oral surfaces has been reported yet. In *in vitro* studies, adherence and interaction of probiotics with existing biofilms seems necessary for any change to occur. Thus, it may be relevant to study both saliva, supra- and subgingival occurrence of exogenous bacteria, since the salivary microbiota might not be the best representative of the composition of dental/denture oral biofilms.

One might question whether the ability of a probiotic strain to permanently colonise the oral cavity would be a desirable trait. Prolonged adhesion and persistence could favour a long-lasting probiotic prophylaxis, which in turn would not require repeated application, thus reducing costs of consumption and treatment. On the contrary, the integration of a probiotic into an existing indigenous niche might disrupt its balance and cause an ecological shift towards more acidogenic microbiota (41). Moreover, the most often employed probiotics are lactic acid bacteria, which have acidogenic potential owing to the production of organic acids thus potentially posing a risk of caries progression (42). So, far the lack of permanent colonisation is reassuring with regard to the safety of dietary probiotics.

There was no obvious effect of consumption of the probiotic product, Yakult, on bacteria associated with malodour, such as facultative and obligate anaerobic species. No black pigmented periodontal pathogens were recovered from any sample. According to the literature, species such as *Aggregatibacter actinomycetemcomitans*, *Porphyromonas gingivalis* and *Prevotella intermedia* can be found in the edentulous mouth despite absence of teeth and periodontal pockets (43). Recently, Van Assche et al., (44) have reported that full-mouth tooth extraction does not eradicate periodontopathogens but result in its reduction. *P. gingivalis* has been found in saliva, periodontal pockets and mucous membranes of edentulous individuals with history of periodontal disease (45).

*Streptococcus mutans* levels were also very low and no change in numbers isolated was found. The ratio of acidogenic to non-acidogenic microorganisms did not change despite introducing an additional acidogenic (*Lactobacillus*) species. This observation suggests that the presence of LcS did not alter the natural acidogenicity of microbial ecosystem. Indeed the microbiota in denture wearers is already characterised by the presence of pH-lowering microorganisms, such as streptococci, lactobacilli and yeast.

There is very limited evidence of any beneficial effect of probiotics on oral *Candida* spp. No significant change in numbers of salivary or tongue plaque *Candida* in colonised individuals was observed throughout the study. Ahola et al., (46) investigated the effect of 3-week consumption of cheese containing three different probiotic bacterial strains on cariogenic microbiota, including salivary yeast count in healthy young adults. There was no significant difference in concentration of yeast between placebo and treatment group. However, consumption of probiotic cheese seemed to reduce the risk of the highest levels of salivary yeast counts. In another study by the same group (47), probiotic cheese was given to elderly participants (age 70–100 years) over a 16-week intervention period. It was found that probiotic treatment reduced the prevalence and risk of high yeast counts (≥ 10^4^ CFU ml^−1^) by 32 and 75%, respectively, in contrast to control group where this prevalence increased. Recently, a group from São Paulo investigated in a randomised double-blind, placebo-controlled intervention study the effect of *L. rhamnosus* and *Lactobacillus acidophilus* probiotic species in denture wearers carrying oral *Candida* (48). Participants applied the probiotic bacteria in a form of powder contained in gel capsules over the maxillary denture, daily for 10 weeks. The authors reported a significant reduction of *Candida* spp. count in 79% of the patients from the experimental group. The greatest yeast prevalence was of *C. albicans* (37%), followed by *C. guilliermondii* (26%), *C. glabrata* and *C. tropicalis* (14% each). This study demonstrated the potential use of probiotics in reducing the prevalence of various species of *Candida*, thus a possible preventative means against oral yeast infections.

Results described above agree with findings from our previous study investigating the effect of consumption of LcS as Yakult on oral health in dentate people. In both study groups, participants were healthy and did not have yeast infection, with numbers of candida generally being below the level of detection.

In this study, we noted changes in responses regarding perceived quality of life related to oral health, measured with modified OHIP-14 questionnaires before and after study. Overall, there was an improvement in the post-study responses but no significant difference was found. Since the intervention study was carried out in a working dental clinic during scheduled appointments, the recruited participants were patients receiving dental treatment. It is possible that the fact of receiving a new set of well-fitted dentures at the end of the treatment, which coincided with the study completion, had influenced the responses associated with physical pain, such as experience of ‘achy mouth’. Pain experienced by denture wearers is frequently caused by badly fitted prostheses, which can be improved with a correct fit. An improvement in the post-treatment OHIP responses in denture wearers has been reported previously and was related to the treatment itself (as in receiving new denture) (49). It is therefore difficult to establish whether 4-week consumption of LcS had an effect on the improved OHIP scores. Perhaps, taking part in the study itself provided patients with feeling of usefulness or of being the centre of attention. Reduced feelings of embarrassment and self-consciousness about malodour were also reported in our study. It can be speculated that information about oral health and denture hygiene provided during the trial, as well as the opportunity to discuss denture-related problems with the study team or dental practitioner, might have made the topic of oral odour more familiar and thus manageable.

One obvious limitation of this trial was low sample size. This was due to difficulties in recruitment of suitable participants, since many patients approached did not fit the inclusion criteria. This problem was encountered previously during the first running of the trial. Despite modifications to the study protocol (reduced number of performed tests) and inclusion criteria (smokers included), the number of suitable and willing patients was again low. Moreover, the study was conducted on NHS patients attending their scheduled dental appointments at a local dental hospital, where time constraint was a major drawback.

## Conclusion

In conclusion, short term (4-week) consumption of the recommended daily intake of the probiotic drink, Yakult, containing a minimum of 6.5×10^9^ viable LcS per 65 ml bottle did not significantly affect the resident oral microbiota in the study group of complete denture wearers. The probiotic strain (LcS) was, however isolated from saliva, tongue and denture plaque during the consumption phase and up to 7 weeks of washout. The probiotic strain transiently colonised the oral cavity but was recovered at relatively low numbers. Indeed, in a healthy, well balanced oral microbiota no significant changes would be desirable; the current results provided an insight into the potential use of probiotics in edentulous denture wearers. Future work could include a larger sample group and concentrate on the less healthy denture-wearing population, such as individuals with denture-associated stomatitis, characterised by high numbers of yeast, or dentures harbouring opportunistic pathogens, where transient probiotic colonisation might have an effect.
